# Is It Possible to Improve the Perceived Quality of Life of Overweight or Obese Older People through a Multicomponent Physical Exercise Program?

**DOI:** 10.3390/bs14070618

**Published:** 2024-07-21

**Authors:** Yazmina Pleticosic-Ramírez, Víctor Arufe-Giráldez, Josune Rodríguez-Negro, Marcos Mecías-Calvo, Rubén Navarro-Patón

**Affiliations:** 1 Departamento de Salud, Universidad Internacional Iberoamericana, Campeche 24560, Mexico; yazmina.pleticosic@doctorado.unini.edu.mx; 2 Facultad de Educación, Pedagogía en Educación Física, Universidad San Sebastián, Lientur 1457, Concepción 4080871, Chile; 3 Facultad de Ciencias de la Educación, Universidad de A Coruña, 15008 A Coruña, Spain; v.arufe@udc.es; 4 Department of Didactics of Musical, Plastic and Corporal Expression, Faculty of Education, University of the Basque Country (UPV/EHU), 48940 Leioa, Spain; josune.rodriguez@ehu.eus; 5 Research Unit of School Sport, Physical Education and Psychomotricity, University of A Coruña, 15008 A Coruña, Spain; 6 Facultade de Formación do Profesorado, Universidade de Santiago de Compostela, 27001 Lugo, Spain; ruben.navarro.paton@usc.es

**Keywords:** quality of life, elderly, ageing, perception, WHOQOL-BREF physical activity

## Abstract

Multicomponent exercise is a physical exercise modality in which various physical qualities (strength, cardiorespiratory endurance, flexibility, and balance) are developed with an equal distribution of volume in the same session (approximately 60 min) and that has been little explored in improving the quality of life of older adults. The aim of this study was to verify the effect of multicomponent training on self-perceived quality of life in Chilean overweight or obese older people. To this end, a quasi-experimental study with a control group was designed to evaluate self-perceived Quality of Life using the World Health Organization Quality of Life, brief version [Overall Quality of Life (OQOL); Overall Health (OH); Physical Health (PH); Psychological Health (PsH); Social Relations (SR); Environment (E)]. Seventy overweight or obese people aged between 60 and 86 years participated (M = 73.15; SD = 5.94) and were randomized into a control group (CG, n = 35) and an experimental group (EG, n = 35). The results in the EG (pre vs. post-intervention) indicated that there were statistically significant differences in OQOL (*p* = 0.005), OH (*p* = 0.014), PH (*p* < 0.001), PsH (*p* < 0.001), E (*p* = 0.015), and SR (*p* < 0.001) which were not found in the CG in any of the variables (*p* > 0.050) except in SR (*p* < 0.001). Regarding sex, post-intervention differences were only found between CG and EG in women in OQOL (*p* = 0.002), PH (*p* < 0.001), PsH (*p* = 0.003), and SR (*p* < 0.001), but not in OH or E (*p* > 0.050). These differences were not found among men in any of the variables (*p* > 0.050). As a conclusion, we can say that a multicomponent physical exercise program applied for 6 months significantly improves the perception of OQOL, OH, PH, PsH, SR, and E in overweight or obese older people. This perception is greater in men than in women.

## 1. Introduction

Nowadays, in developed countries, reaching an advanced age is no longer exceptional [1]. The demographic transformation towards an aging society is perceived as a sociodemographic change that has been increasing in recent decades [2]. Ageing brings with it physical limitations or chronic diseases that cause a lack of full well-being in this sector of the population [3,4]. However, well-being can be improved through the practice of physical exercise (PE) [5], which consequently causes an increase in the self-perceived quality of life in older adults [6]. Quality of life refers to an individual’s perception of his or her position in life in the context of the culture and value system in which he or she lives and in relation to his or her goals, expectations, standards, and concerns [7,8].

Relating PE to quality of life, Vázquez et al. [5] found a positive association between quality of life and higher levels of physical activity, as well as lower levels of depression and dependence. Bouaziz et al. [9] mention that regular exercise has been shown to have many health benefits, positively impacting quality of life.

Health-related quality of life has been studied through the SF-36 questionnaire in previous research, where significant effects were obtained on physical performance and quality of life [10]; in the mental component and mental health subscale with the practice of HITT [11]; pain reduction, social and vitality improvements with the practice of moderate intensity aquatic training; improving vitality and health status with HIIT aquatic training [12]; improvement in general health, physical functioning, mental health, and vitality with resistance training [13]; and improvement in health status, vitality, and social aspects with multicomponent aquatic training [14]. Previous studies related to improvements through multicomponent exercise have analyzed improvements in BMI [15], functional capacity, or physical capacity [16], among others. Finally, the scientific evidence to date, including the WHOQOL-BREF questionnaire and multicomponent physical exercise, to evaluate quality of life in older adults is scarce or has been carried out only in women [17,18] or only to evaluate cognitive functions [19].

Multicomponent exercise is a PE modality in which various physical qualities (strength, cardiorespiratory endurance, flexibility, and balance) are developed with an equal distribution of volume in the same session (approximately 60 min) [20] and that has been little explored in improving the quality of life of older adults.

For all of the above, and given the lack of evidence on the effect of multicomponent PE on quality of life in older adults, the objective of this study was to verify the effect of multicomponent training on self-perceived quality of life in older Chilean overweight or obese people and whether these effects are the same in men and women.

## 2. Materials and Methods

### 2.1. Study Design

For this study with a quasi-experimental design with pre- and post-test measures, with a control group [21], the World Health Organization questionnaire (WHOQOL-BREF) [22] was used to evaluate quality of life, adapted to the Chilean adult population [23] (i.e., Overall Quality of Life (OQOL); Overall Health (OH); Physical Health (PH); Psychological Health (PsH); Environment (E); Social Relations (SR)) according to the group (control vs. experimental), and this was stratified according to gender (man vs. woman).

### 2.2. Participants

A total of 153 individuals who were overweight or obese and were 60 years of age or older—59 men and 94 women—were invited to take part in this study. A convenience sample was provided of members of clubs affiliated with the Regional Federation of Community Unions of the Elderly in the Biobío area of Concepción, Chile. The following criteria had to be met in order to be considered for inclusion: (a) being 60 years of age or older; (b) being overweight or obese people according to WHO criteria [24]; (c) not having a medical condition that would prevent them from taking part in the tests or intervention program; (d) being physically independent; (e) signing an informed consent form.

After fulfilling all the requirements for admission, 70 participants were randomly assigned to one of two groups: the experimental group (EG, n = 35; 28 women/7 men) or the control group (CG, n = 35; 33 women/2 men).

### 2.3. Instruments

#### 2.3.1. Sociodemographic Data

The data on the variables age (years) and sex (male/female) were self-reported.

#### 2.3.2. Anthropometric and Body Composition Measurements

For anthropometric and body composition measurements, the protocol of the International Society for the Advancement of Kinanthropometry (ISAK) [25] was used for both body mass and height. These two allowed us to determine the degree of obesity through the body mass index (BMI) with the formula [weight kg/height m^2^], following the WHO measurements [24].

The height measurement was performed with the portable SECA 206 stadiometer in the maximum extension position, placing the square firmly on the Vertex, compressing the hair as much as possible, and asking the person to inhale deeply and hold their breath before the subject evaluated exhaled [25].

Body mass was calculated using the Omrom HBF-514C equipment. Weight was evaluated with minimal clothing, checking that the scale was at zero. These measurements were routinely performed in the morning, twelve hours after the last meal [25].

#### 2.3.3. Adapted World Health Organization Quality of Life Questionnaire (WHOQOL-BREF)

The self-administered questionnaire of the World Health Organization (WHOQOL-BREF) [22] was used to evaluate the quality of life adapted to the Chilean older people population [23]. This self-administered questionnaire is composed of a general question on quality of life (“How would you rate your quality of life?”), a question on satisfaction with your health status (“Are you satisfied with your health?”), and 24 items grouped into four dimensions: Physical Health (e.g., “To what extent do you think that physical pain prevents you from doing what you need?”), Psychological Health (e.g., “To what extent do you feel that Does your life have meaning?”), Social Relationships (e.g., “To what extent are you satisfied with your interpersonal relationships?”), and Environment (e.g., “To what extent are you satisfied with the conditions of the place where you live?”). The items are scored on a five-point Likert 1 scale with five different formats (1 = “very bad” to 5 = “very good”, 1 = “very dissatisfied” to 5 = “quite satisfied”, 1 =not at all to 5= “a lot”, 1= “very little” to 5= “very good”, 1= “never” to 5= “always”).

#### 2.3.4. Intervention Program

**Figure 1:**
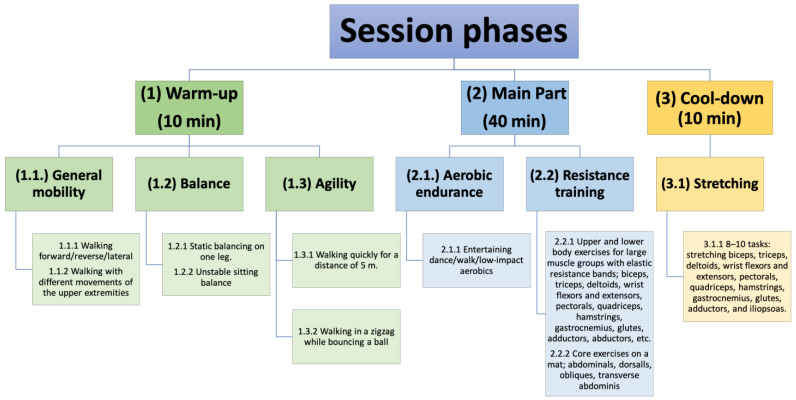
Phases and contents of the sessions and types of exercises of the multicomponent program.

The EG participated in a multicomponent physical exercise program. This is defined as a type of training that incorporates different elements, such as cardiovascular training, coordination, strength, balance, and flexibility, in a single exercise session [26] which was taught by the main researcher, who has 15 years of experience in the field of Physical Education. The program lasted 6 months, with 2 sessions per week, each lasting 60 min. Each session was organized as can be seen in Figure 1. The CG participants continued with their daily lives without modifying their habits or participating in any physical exercise program.

### 2.4. Procedure

Contact was made first with the management of the clubs of the Regional Federation of Community Unions of older people in the Biobío region of the city of Concepción (Chile), and the objective of the study was explained to them. After management approval, an invitation letter was sent to potential participants for an informational meeting to explain the aim and purpose of the study, the procedure, and their voluntary participation, as well as the confidentiality statement.

After the participants signed the informed consent, the necessary sociodemographic data (age and sex) were self-reported and the participants were randomly assigned to EG and CG. The anthropometric measurements (i.e., height, weight, and BMI following the formula BMI = weight kg/height m^2^) and the self-administered questionnaire were collected prior to the start of the intervention. The survey lasted approximately 15 min. Once the initial data was collected, the intervention program was applied to the EG. Once the intervention period was over, data on quality of life were collected using the WHOQOL-BREF questionnaire for both groups (CG and EG) within one week after completing the intervention program. All research was carried out in accordance with the Declaration of Helsinki. The research protocol was sent and approved by the Ethics Committee of the Universidad Internacional Iberoamericana on 22 June 2022, being approved and registered in file number CR-163.

### 2.5. Statistical Analysis

The IBM SPSS Statistics for Windows program, version 25.0, was used to statistically analyse the data in this study. Measures of central tendency (mean and standard deviation) are used to present the results of the quantitative variables (Overall Quality of Life (OQOL), Overall Health (OH), Physical Health (PH), Psychological Health (PsH), Environment (E), Social Relations (SR), BMI, height, weight, and age); percentages and frequencies are used to present the results of the qualitative variables (sex and degree of overweightness or obesity). The Kolmogorov–Smirnov test was performed to confirm the normality of the data. First, for each dependent variable under investigation, the descriptive statistics (mean and standard deviation) were determined. Second, an independent sample *t*-test was used to determine whether the groups (the experimental group and the control group) were equivalent in terms of age and anthropometry (BMI, height, weight), the Chi square test was used to determine whether the groups were equivalent in terms of the participants’ sex and degree of obesity or overweightness, and finally, an independent samples T test was used to check for changes in BMI, height, and weight. Using Time as a repeated measures factor (i.e., Time (pre-test vs. post-test), Group (Control group vs. Experimental group), and Sex (man vs. woman)) to analyse the potential main effect of these factors on the variables of the WHOQOL-BREF questionnaire and their interaction using the statistic Bonferroni, a three-factor ANOVA (time x group x degree of overweight or obesity) was conducted after the six-month intervention. The eta squared (η2) was used to calculate the effect size. 

## 3. Results

The sample was divided into two analysis groups, the CG (n = 35), with a mean age of 72.54 years (SD = 5.55), and the EG (n = 35), with a mean age of 73.77 years (SD = 6.32). Regarding the sex variable, 87% of the participants were women (n = 61) and 13% men (n = 9), distributing 33 women and 2 men in the CG and 28 women and 7 men in the EG. The baseline characteristics of the sample (Table 1) indicate that there were no statistically significant differences in any of the variables [i.e., mean age (*p* = 0.391); gender (*p*= 0.075); average height (*p* = 0.685); average weight (*p* = 0.443); BMI (*p* = 0.215) and degree of overweight-obesity (*p* = 528)] between the CG and EG.

The normality test revealed that the data followed a normal distribution [i.e., OQOL (*p* = 0.121), OH (*p* = 0.243), PH (*p* = 0.516), PsH (*p* = 0.412), E (*p* = 0.288), and SR (*p* = 0.300)].

### 3.1. CG and EG Pre-Intervention Comparison

The results before the intervention indicated that there were no statistically significant differences in any of the variables studied [i.e., OQOL (*p* = 0.084), OH (*p* = 0.783), PH (*p* = 0.175), PsH (*p* = 0.732), SR (*p* = 0.715), and E (*p* = 0.705)] in the comparison between the CG and EG (Table 2).

**Figure 2:**
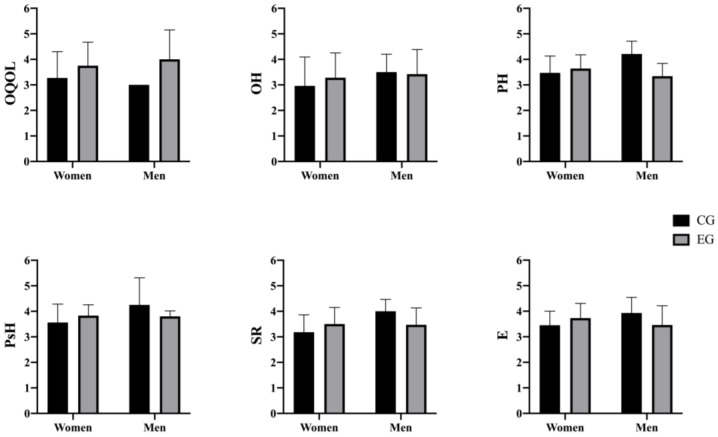
Pre-test differences between CG and EG. CG: control group; EG: experimental group; OQOL: Overall Quality Of Life; OH: Overall Health; PH: Physical Health; PsH: Psychological Health; SR: Social Relations; E: Environment.

Depending on sex (Figure 2), the results indicated that there were no previous statistically significant differences between women in the CG and women in the EG in any of the variables studied [i.e., OQOL (*p* = 0.067); OH (*p* = 0.246); PH (*p* = 0.272); PsH (*p* = 0.085); SR (*p* = 0.067); E (*p* = 0.062)], nor between men from the CG and men from the EG [i.e., OQOL (*p* = 0.216); OH (*p* = 0.933); PH (*p* = 0.077); PsH (*p* = 0.360); SR (*p* = 0.330); E (*p* = 0.318)]. For all these reasons, the groups were equivalent with respect to all the variables studied.

### 3.2. Control Group Results

The pre- and post-intervention results of the CG (Table 1) indicated that there were statistically significant differences in OQOL [F(1, 66) = 4.423, *p* = 0.039, η2 = 0.063, 95% CI −1.093, −0.028], with the scores being lower before than after 6 months, but not in the rest of the variables [i.e., OH (*p* = 0.737); PH (*p* = 0.668); PsH (*p* = 609); SR (*p* = 0.724); E (*p* = 0.827)].

**Figure 3:**
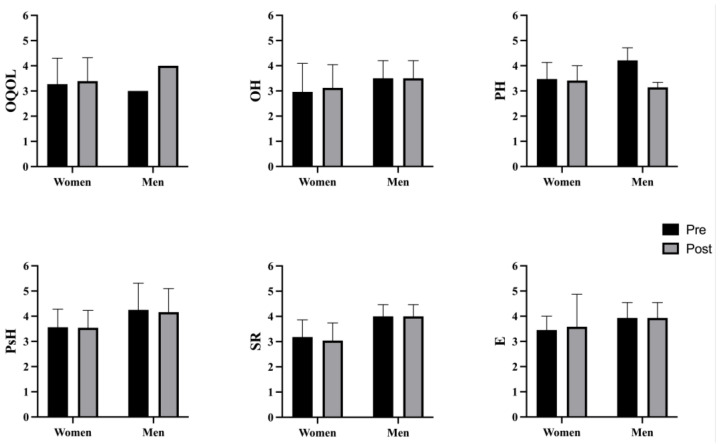
Pre-post-test sex differences in the CG. CG: control group; OQOL: Overall Quality Of Life; OH: Overall Health; PH: Physical Health; PsH: Psychological Health; SR: Social Relations; E: Environment.

There were no statistically significant differences in any of the variables studied in the pre–post comparison in the CG, depending on sex (Figure 3), nor in women [i.e., OQOL (*p* = 0.345); OH (*p* = 0.164); PH (*p* = 0.071); PsH (*p* = 0.676); SR (*p* = 0.143); E (*p* = 0.363)], nor in men [i.e., OQOL (*p* = 0.058); OH (1.00); PH (*p* = 0.288); PsH (*p* = 0.671); SR (*p* = 1.00); E (*p* = 1.00)].

### 3.3. Experimental Group Results

The pre- and post-intervention results of the EG (Table 1) indicated that there were statistically significant differences in all the variables of the questionnaire (i.e., OQOL [F(1, 66) = 11.995, *p* < 0.001, η2 = 0.154, 95% CI −0.845, −0.227]; OH [F(1, 66) = 9.894, *p* = 0.002, η2 = 0.130, 95% CI −0.671, −0.150]; PH [F(1, 66) = 24.187, *p* < 0.001, η2 = 0.268, 95% CI −0.595, −0.252], PsH [F(1, 66) = 48.737, *p* < 0.001, η2 = 0.425, 95% CI −0.524, −0.291]; SR [F(1, 66) = 12.557, *p* = 0.001, η2 = 0.160, 95% CI −0.642, −0.179] and E [F(1, 66) = 6.321, *p* = 0.001, η2 = 0.087, 95% CI −0.793, −0.091]).

**Figure 4:**
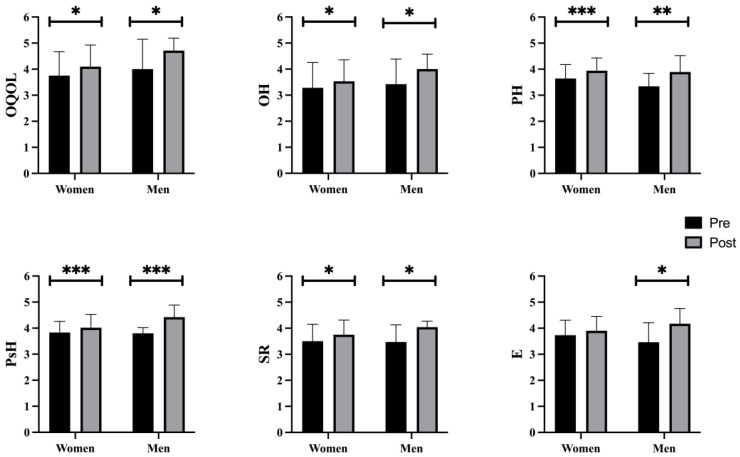
Pre-post-test sex differences in the EG. EG: experimental group; OQOL: Overall Quality Of Life; OH: Overall Health; PH: Physical Health; PsH: Psychological Health; SR: Social Relations; E: Environment. Note: * *p* < 0.05 different between Pre- vs. Post-test; ** *p* = 0.001 different between Pre- vs. Post-test; *** *p* < 0.001 different between Pre- vs. Post-test.

In the comparison of pre- and post-intervention in the EG (Figure 4), there were statistically significant differences in all the variables studied between women (OQOL [F(1, 66) = 6.664, *p* = 0.012, η2 = 0.092, 95% CI −0.633, −0.081; OH [F(1, 66) = 4.582, *p* = 0.036, η2 = 0.065, 95% CI −0.483, −0.017]; PH [F(1, 66) = 14.764, *p* < 0.001, η2 = 0.183, 95% CI −0.450, −0.142]; PsH [F(1, 66) = 14.139, *p* < 0.001, η2 = 0.176, 95% CI −0.301, −0.092]; SR [F(1, 66) = 5.1816, *p* = 0.019, η2 = 0.176, 95% CI −0.457, −0.043], except in E (*p* = 0.285); and between men (OQOL [F(1, 66) = 6.664, *p* = 0.012, η2 = 0.092, 95% CI −1.267, −0.162]; OH [F(1, 66) = 5.985, *p* = 0.017, η2 = 0.083, 95% CI −1.038, −0.105]; PH [F(1, 66) = 12.797, *p* = 0.001, η2 = 0.162, 95% CI −0.859, −0.243]; PsH [F(1, 66) = 35.107, *p* < 0.001, η2 = 0.347, 95% CI −0.828, −0.410]; SR [F(1, 66) = 7.596, *p* = 0.008, η2 = 0.347, 95% CI −0.985, −0.157], and E [F(1, 66) = 5.160, *p* = 0.026, η2 = 0.073, 95% CI −1.342, −0.086]).

### 3.4. CG vs. EG Post-Intervention Results

There were statistically significant differences post-intervention results between the CG and EG (Table 1) only in OQOL [F(1, 66) = 3.954, *p* = 0.005, η2 = 0.057, 95% CI −1.430, −0.003]. There were no statistically significant differences in OH (*p* = 0.212), PH (*p* = 0.552), PsH (*p* = 0.155), SR (*p* =0.149), or E (*p* = 0.501).

**Figure 5:**
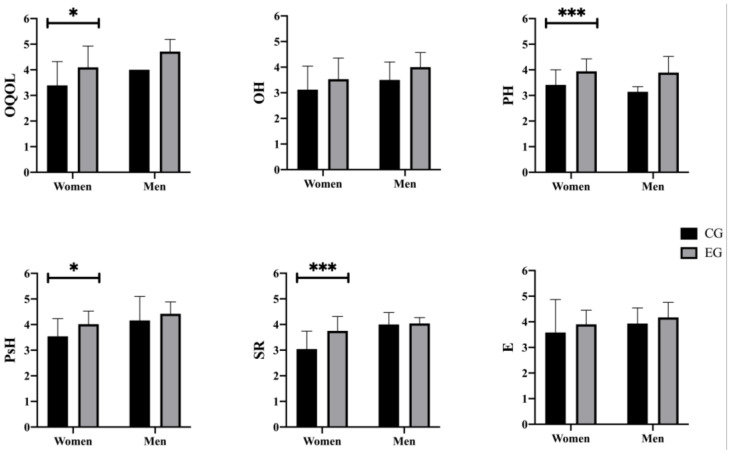
Post-test sex differences between CG and EG. CG: control group; EG: experimental group; OQOL: Overall Quality Of Life; OH: Overall Health; PH: Physical Health; PsH: Psychological Health; SR: Social Relations; E: Environment. Note: * *p* < 0.05 different between Pre- vs. Post-test; *** *p* < 0.001 different between CG vs. EG Post-test.

In the post-intervention comparison between groups (CG vs. EG) (Figure 5), after carrying out the stratified analysis by gender and analyzing their interaction, there were statistically significant differences in almost all the variables studied between women (OQOL [F(1, 66) = 10.597, *p* = 0.002, η2 = 0.138, 230 95% CI −1.151, −0.276]; PH [F(1, 66) = 13.558, *p* < 0.001, η2 = 0.170, 95% CI −0.808, −0.098]; PsH 231 [F(1, 66) = 9.355, *p* = 0.003, η2 = 0.124, 95% CI −0.800, −0.168]; and SR [F(1, 66) = 20.080, *p* < 232 0.001, η2 = 0.233, 95% CI −1.026, −0.393], except in OH (*p* = 0.066) and E (*p* = 0.209).

Regarding men, no statistically significant differences were found in any of the variables studied [i.e., OQOL (*p* = 0.300), OH (*p* = 0.472), PH (*p* = 0.583), PsH (*p* = 0.598), SR (*p* = 0.924), and E (*p* = 0.762)].

## 4. Discussion

The aim of the present study was to determine the effect of a six-month multicomponent PE program on the perceived quality of life of older Chilean overweight or obese people and whether these effects are similar in women and men. At a general level, the results obtained indicate the beneficial effects of practicing PE in older people [5,27], specifically highlighting that a multicomponent PE program is associated with improvements in the perceived quality of life in older Chilean overweight or obese adults [15,28].

Before the intervention, the CG and the EG presented similar scores in QOL, since there were no statistically significant differences in any of its components (i.e., OQOL; OH; PH; PsH; SR; E), neither globally [28,29] nor based on sex [30]. These results could be due to the fact that the sample was pulled from the same population source and had similar ages and BMIs.

After the intervention using the multicomponent PE program, significant improvements in OQOL were found when comparing CG versus EG. These improvements in the scores given by the EG may be related to the fact that a PE program, such as the one implemented in this research, produces changes and improvements in the functional abilities of the participants [17] and, consequently, in improving the perception of quality of life. These results are consistent with the results obtained by Tricco et al. [31] and Villareal et al. [32], who observed a positive correlation between the increase in performance in general EF with the increase in quality of life in older people with obesity after six months of intervention, as in our case. Depending on sex, the results indicated that there were significant differences between women in the CG and EG after the intervention in OQOL, PH, PsH, and SR. These results may be associated with and related to what was reported in previous studies, which indicates that regular PE can improve results beyond physical health and generate greater social interaction [33]. However, no significant differences were found between men in both groups.

After the intervention using multicomponent PE in the EG, there were significant improvements in all components of the perceived quality of life evaluated with the WHOQOL-BREF questionnaire (i.e., OQOL; OH; PH; PsH; SR; E), globally [34] and in both men [35,36] and women [17], which did not occur in the CG. This improvement in quality of life may be related to participation in PE programs where muscle strength is increased, contributing to the performance of more tasks of daily living with less effort and help. Consequently, participants perceive an improvement in levels of health and physical and psychological well-being, improving social interaction by perceiving that they are more independent [37]. Our results are similar to those reported by Whitehurst et al. [38], who, after applying a multicomponent PE program and evaluating quality of life with the short form health survey (SF-36), observed an improvement in the perceived quality of life, as well as those reported by Maung et al. [34] or Atad and Caspi [39], who showed that performing 2.5 h of PE per week not only produced better physical health but was also associated with a better perceived quality of life.

Regarding the CG, once the intervention period was over, the scores of the different dimensions of quality of life of the components studied were maintained and, in some cases, decreased. These results are consistent with those obtained by Resende-Neto et al. [17] since the improvement in the perception of quality of life occurs alongside participation in systematic PE programs, which was not carried out by this group.

## 5. Limitations

This research has limitations that we want to note; the first of them is the use of a self-reported questionnaire, which can produce biases in the participants’ responses. Another limitation is the size of the sample, due to its limited number and its selection for convenience and the possibility of access. Regarding the sample, it should also be noted that the small number of male participants is very limited due to the characteristics of participation in physical activity programs, and therefore, we cannot extrapolate the results to this group. Thus, more studies would be necessary in which it would be possible to increase the sample size in this regard.

Finally, we must indicate that a long-term follow-up was not carried out to verify whether this multicomponent physical exercise program maintains its long-term effect on the perceived quality of life of overweight or obese older people.

## 6. Conclusions

As conclusions of this study, we can say that a multicomponent physical exercise program, applied for 6 months, produces significant improvements in self-perceived quality of life and global health, as well as physical and psychological health, social relationships, and the environment, in overweight or obese older people. However, no improvements occurred in older people who did not participate in this multicomponent exercise program.

We can also conclude that a multicomponent physical exercise program produces a greater perception of quality of life in men compared to women in all the variables studied except in physical health, where women give higher scores.

## Figures and Tables

**Table 1:** Sample characterization.

	Control Group	Experimental Group
**Variables**		
**Average age (years)**	72.54 ± 5.55	73.77 ± 6.32
**Sex**		
Man	2 (72.2%)	7 (27.8%)
Woman	33 (27.8%)	28 (72.2%)
**Average height (m)**	1.538 ± 7.16	1.530 ± 9.16
**Average weight (kg)**	72.51 ± 11.99	74.80 ± 12.75
**Average BMI (kg/m^2^)**	30.71 ± 4.075	31.88 ± 3.73
**Degree of overweight-obesity**		
Overweight	17 (24.3%)	11 (15.7%)
Type I Obesity	13 (18.6%)	17 (24.3%)
Type II Obesity	4 (5.7%)	6 (8.6%)
Type III Obesity	1 (1.4%)	1 (1.4%)

Note: Quantitative variables are expressed as mean and standard deviation, and qualitative variables are expressed as frequencies and percentages.

**Table 2:** Pre- and post-intervention results of the control and experimental groups.

Variable	CG Pre (n = 35)	EG Pre (n = 35)	CG Post (n = 35)	EG Post (n = 35)
Quality of Life	3.25 ± 1.01	3.80 ± 0.96	3.42 ± 0.91	4.22 ± 0.80
Overall Health	3.00 ± 1.11	3.31 ± 0.96	3.14 ± 0.91	3.62 ± 0.80
Physical Health	3.51 ± 0.67	3.58 ± 0.54	3.46 ± 0.59	3.93 ± 0.51
Psychological Health	3.60 ± 0.74	3.82 ± 0.39	3.58 ± 0.71	4.10 ± 0.52
Social Relations	3.22 ± 0.69	3.49 ± 0.64	3.09 ± 0.72	3.80 ± 0.52
Environment	3.47 ± 0.55	3.68 ± 0.61	3.60 ± 1.26	3.96 ± 0.56

Note: Data are presented as mean ± standard deviation.

## Data Availability

The datasets presented in this article are not available because they are part of a doctoral thesis which has not yet been defended. Requests to access the datasets should be directed to Y.P.-R.
